# OBO Foundry in 2021: operationalizing open data principles to evaluate ontologies

**DOI:** 10.1093/database/baab069

**Published:** 2021-10-26

**Authors:** Rebecca Jackson, Nicolas Matentzoglu, James A Overton, Randi Vita, James P Balhoff, Pier Luigi Buttigieg, Seth Carbon, Melanie Courtot, Alexander D Diehl, Damion M Dooley, William D Duncan, Nomi L Harris, Melissa A Haendel, Suzanna E Lewis, Darren A Natale, David Osumi-Sutherland, Alan Ruttenberg, Lynn M Schriml, Barry Smith, Christian J Stoeckert Jr., Nicole A Vasilevsky, Ramona L Walls, Jie Zheng, Christopher J Mungall, Bjoern Peters

**Affiliations:** Bend Informatics LLC, 20770 Double Peaks Drive, Bend, OR 97701, USA; Semanticly, 71-75 Shelton Street, London WC2H 9JQ, UK; Knocean Inc., 2-107 Quebec Ave., Toronto, ON M6P 2T3, Canada; La Jolla Institute for Immunology, 9420 Athena Cir, La Jolla, CA 92037, USA; Renaissance Computing Institute, University of North Carolina, 100 Europa Drive, Suite 540, Chapel Hill, NC 27517, USA; Alfred Wegener Institute, Helmholtz Center for Polar and Marine Research, Am Handelshafen 12, Bremerhaven 27570, Germany; Environmental Genomics and Systems Biology, Lawrence Berkeley National Laboratory, 1 Cyclotron Rd., Berkeley, CA 94720, USA; European Bioinformatics Institute (EMBL-EBI), Wellcome Genome Campus, Hinxton CB10 1SD, UK; Department of Biomedical Informatics, University at Buffalo, 77 Goodell St, Buffalo, NY 14203, USA; Centre for Infectious Disease Genomics and One Health, Simon Fraser University, 8888 University Dr, Burnaby, BC V5A 1S6, Canada; Environmental Genomics and Systems Biology, Lawrence Berkeley National Laboratory, 1 Cyclotron Rd., Berkeley, CA 94720, USA; Environmental Genomics and Systems Biology, Lawrence Berkeley National Laboratory, 1 Cyclotron Rd., Berkeley, CA 94720, USA; Biochemistry and Molecular Genetics Department, University of Colorado School of Medicine, PO Box 6511, Aurora, CO 80045, USA; Environmental Genomics and Systems Biology, Lawrence Berkeley National Laboratory, 1 Cyclotron Rd., Berkeley, CA 94720, USA; Department of Biochemistry and Molecular & Cellular Biology, Georgetown University Medical Center, 2115 Wisconsin Avenue NW, Washington, DC 20007, USA; European Bioinformatics Institute (EMBL-EBI), Wellcome Genome Campus, Hinxton CB10 1SD, UK; Department of Biomedical Informatics, University at Buffalo, 77 Goodell St, Buffalo, NY 14203, USA; School of Medicine, University of Maryland, 655 W Baltimore St S, Baltimore, MD 21201, USA; Department of Biomedical Informatics, University at Buffalo, 77 Goodell St, Buffalo, NY 14203, USA; Department of Genetics and Institute for Biomedical Informatics, Perelman School of Medicine, University of Pennsylvania, 3400 Civic Center Blvd, Philadelphia, PA 19104, USA; Biochemistry and Molecular Genetics Department, University of Colorado School of Medicine, PO Box 6511, Aurora, CO 80045, USA; Critical Path Institute, 1730 E River Rd #200, Tucson, AZ 85718, USA; Department of Genetics and Institute for Biomedical Informatics, Perelman School of Medicine, University of Pennsylvania, 3400 Civic Center Blvd, Philadelphia, PA 19104, USA; Environmental Genomics and Systems Biology, Lawrence Berkeley National Laboratory, 1 Cyclotron Rd., Berkeley, CA 94720, USA; La Jolla Institute for Immunology, 9420 Athena Cir, La Jolla, CA 92037, USA

## Abstract

Biological ontologies are used to organize, curate and interpret the vast quantities of data arising from biological experiments. While this works well when using a single ontology, integrating multiple ontologies can be problematic, as they are developed independently, which can lead to incompatibilities. The Open Biological and Biomedical Ontologies (OBO) Foundry was created to address this by facilitating the development, harmonization, application and sharing of ontologies, guided by a set of overarching principles. One challenge in reaching these goals was that the OBO principles were not originally encoded in a precise fashion, and interpretation was subjective. Here, we show how we have addressed this by formally encoding the OBO principles as operational rules and implementing a suite of automated validation checks and a dashboard for objectively evaluating each ontology’s compliance with each principle. This entailed a substantial effort to curate metadata across all ontologies and to coordinate with individual stakeholders. We have applied these checks across the full OBO suite of ontologies, revealing areas where individual ontologies require changes to conform to our principles. Our work demonstrates how a sizable, federated community can be organized and evaluated on objective criteria that help improve overall quality and interoperability, which is vital for the sustenance of the OBO project and towards the overall goals of making data Findable, Accessible, Interoperable, and Reusable (FAIR).

Database URL http://obofoundry.org/

## Introduction

The quantity and complexity of data generated by biological experiments are growing at an unprecedented rate. Ontologies are used to organize, annotate and analyze these data and to harmonize the rich and varied information captured in key biological knowledge bases (1). A major challenge faced by researchers is the large numbers of different overlapping ontologies, varying in quality and completeness, each attempting to cover different aspects of any given domain of interest. For example, BioPortal (2) includes over 800 ontologies and close to 10 million terms as of April 2021 (https://bioportal.bioontology.org/). These challenges are compounded when we consider the fact that many applications require using ‘combinations’ of ontologies. If ontologies are constructed using different principles, they will not work together in a modular, interoperable, and coherent way.

**Figure 1.**
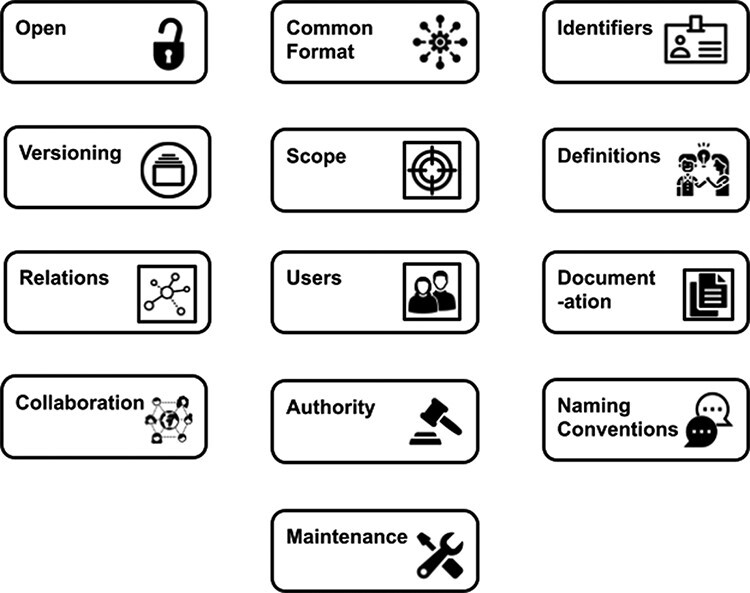
Illustration of the principles around which the OBO Foundry was built.

The Open Biological and Biomedical Ontologies (OBO) project was initiated in the early 2000s, as it became clear that there was a community desire to expand ontologies beyond the scope of the Gene Ontology to tackle biological and biomedical problems more broadly (3). OBO was designed to organize and guide the development of ontologies according to common standards and principles (4), enabling modular composition of ontologies and providing guarantees of technical and scientific quality. One of the mechanisms was a set of principles, which were to be followed by all ontologies within the OBO Foundry (Figure 1). For example, OBO ontologies must be ‘open’, allowing for reuse, and the ontologies should conform to shared standards for how terms are interrelated. Any changes mentioned in this manuscript are in reference to the principles as of the 2007 OBO Foundry manuscript (4). Currently, OBO is governed by a volunteer team consisting of ontology maintainers and stakeholders (the ‘OBO operations committee’), represented by the authors of this manuscript. This team carries out multiple duties, including maintaining the site, stewarding the principles and curating ontology metadata.

Here, we describe our efforts to operationalize the OBO Foundry principles. Working closely with stakeholders across OBO, we have refined the principles, codifying them into operational tests that can be executed automatically at regular intervals. We have implemented a dashboard that provides a matrix view indicating the conformance to each principle for each of the over 150 active ontologies in OBO, allowing drill-down to see complete reports. This work involved significant community effort, working with individual ontologies, and required a wholesale re-curation of ontology metadata across OBO. The results allow both ontology developers and the broader community of users to see the steps each ontology must take to come into conformance.

## Related work

Metadata standards to increase the FAIRness of ontologies are not unique to the OBO Foundry. In the past, work has been done to create the Ontology Metadata Vocabulary to help enable ‘access and reuse of ontologies’ (5). While the Ontology Metadata Vocabulary is not used by the OBO Foundry, many concepts are shared, such as licenses, descriptions and developer contact information. In the more general domain of linked data, there is also the Vocabulary of a Friend, which allows for the description of inter-vocabulary relationships (6). More recently, AgroPortal studied common metadata practices and used their results to build a new metadata model that harmonized these existing practices (7). Finally, Matentzoglu *et al.* published the Minimum Information for Reporting an Ontology guidelines that took input from the OBO Foundry principles, among other projects, to facilitate consistency in ontology documentation (8).

## Results

### Capturing consistent ontology metadata in the OBO registry

OBO considers two sources of information for each ontology project: the ontology itself and metadata provided by the ontology maintainers stored in the OBO registry (http://obofoundry.org/). In the future, it may be better to contain all metadata in the ontology file alone, but we currently think it necessary to use two separate resources for the following reasons

1. To identify the most current version of the ontology.
2. To provide details about how the ontology fits into the registry, which are not details about the ontology file artefact itself.
3. To allow for change in the point of contact (or other metadata) without needing to release a new version of the ontology.

To automate the evaluation of principles across OBO ontologies, we first wanted to ensure that the OBO registry entries accurately and consistently captured the minimal information listed in Table 1. These metadata are the bare minimum that the OBO Foundry maintainers feel are necessary to help users access and reuse the ontology. This includes basic details like the ontology title and a point of contact. As noted in a survey from the 2020 FAIRsFAIR Minimum Metadata Schema for Semantic Artefacts Workshop (9), when searching for and selecting an ontology, participants desired to know both if an ontology is ‘actively maintained’ and its ‘natural language description’. These are reflected in our minimal model as ‘activity status’ and ‘description’, respectively. Many registry metadata files include additional items that were mentioned in this survey, such as domain, uses and issue trackers.

**Table 1.** Minimal ontology metadata captured in the OBO registry

Field	Definition	Example	Automated validation
Title	Full name of the ontology	Ontology for Biomedical Investigations	Must be present
id	Abbreviation of the ontology’s name used as the exclusive namespace for the ontology	Obi	Lowercase, alphanumeric, no spaces
homepage	Website where a user can find information about the ontology	http://obi-ontology.org/	Must be in a URL format
contact.label	Name of a person responsible for the ontology	Bjoern Peters	Must not contain ‘@’ and only have one label
contact.email	Email address of the person responsible for the ontology	bpeters@lji.org	Must be in email format and only have one email
products.id	Name of the canonical ontology file (id.owl). Additional products may include ontology subsets, bridge files, etc.	obi.owl	Format enforced
description	Concise free text description of the scope of the ontology	An integrated ontology for the description of life science and clinical investigations	Must be present
license.label	Name of license	CC-BY 4.0	Must correspond to the title of the license.url
license.url	URL of license	https://creativecommons.org/licenses/by/4.0/	Must be in a URL format
activity status	Indicates the development status of the ontology	active	Must be one of ‘active’, ‘inactive’ or ‘orphaned’
obsoletion status	Indicates if the ontology has been declared obsolete by the original developers	false	Must be either ‘true’ or ‘false’

The OBO registry has grown from a short and simple list of a dozen ontologies to a comprehensive resource for metadata on more than 150 active projects. To ensure that the information in the OBO registry was up to date, we emailed the indicated contact persons for each ontology. If no response was obtained, we used personal contacts as well as searches on PubMed and Google to try to find alternative contacts. When we began this work in 2018, we found that out of 201 ontologies, 145 were under current active development, 5 were in use but not being actively developed, 45 were obsoleted and for 6, no contact person could be identified, making them ‘orphaned’. For the active ontologies, we asked the developers to confirm and update fields in the OBO registry, specifically the ontology title, homepage, contact, description and license. This resulted in a total of over 60 updates to OBO registry metadata, most of which were additions of previously missing information.

To ensure that the OBO registry records will be kept up to date over time, we created a lightweight system for collaboratively curating and updating these records. Metadata files are stored in a structured format under version control in a repository within the OBO GitHub organization. This allows both ontology maintainers and members of the core OBO team to make suggestions via GitHub pull requests. These metadata are visible to the community via the OBO registry website or in computable format (YAML and JSON-LD) and are used in order to evaluate an ontology according to the newly operationalized principles. As of May 2021, there have been 3045 commits by 113 developers to the repository, demonstrating that this system is adequate for broad use by the OBO community. The end result of this process is consistent and quality-controlled metadata for each ontology, and a procedure for ensuring these can be easily kept up to date by the community.

### Defining operating principles for OBO ontologies

We took the original set of OBO principles and, for each one, refined them until we had arrived at a more crisply stated operational procedure. These principles were always envisioned as being evolutionary and have been reworded and added to throughout the years. It is true that many of these principles are broken by many of the ontologies in the OBO Foundry. Conforming to all principles is not currently a requirement to be included in the list of OBO ontologies. Rather, by listing the conformance to different principles, we hope to motivate groups to modify their ontologies in order to improve their compliance.

For example, the first principle of OBO is that the ontology is ‘open’. However, there were no specific recommendations on the licensing terms that would meet that goal, or of how the license should be stated. Some ontologies included license information on their home page, others embedded it in their ontology metadata. After community discussions, we agreed that ontologies could be considered ‘open’ for the purposes of OBO if they used the Creative Commons Attribution (CC BY) license 3.0 or later or if they were in the public domain using the Creative Commons CC0 declaration. Both of these options conform to the spirit of the original principle of openness and were already adopted widely by a majority of OBO ontologies as well as many community projects. Next, we settled on a convention on how the license should be stated and decided on the use of the widely accepted Dublin Core Terms (10) ‘license’ property (‘dcterms:license’) in the ontology file metadata in addition to a declaration of the license in the OBO registry entry. These conventions allow checking for the presence of an ‘Open’ license computationally, in both the ontology file itself and the information contained in the OBO registry.

Following the same process for each principle, Table 2 lists how each principle is now encoded with a succinct summary of the principle using ISO MUST/SHOULD language (11) (https://tools.ietf.org/html/rfc2119), and a description of the automated check being performed. A more detailed description of each principle is linked to, which includes a description of the Purpose (what the principle is intended to achieve), ‘Recommendations’ for ontology developers describing how they should best conform to the principle, examples of ‘Implementation’ of the principle, ‘Counter examples’ showing how an ontology could fall short of conformance to the principle and ‘Criteria for review’ that spell out what a human reviewer should be looking for in an ontology in order to judge if it adheres to the principle or not. Each principle has a corresponding issue related to its automated validation on the public GitHub repository (https://github.com/OBOFoundry/OBOFoundry.github.io) in which further questions and discussions are tracked (Table 3). Additionally, there is a continuous review in bi-weekly conference calls of new questions and the need to update the wording of principles. At the same time, anyone is able to asynchronously comment on the process by adding their comments to the relevant GitHub issue.

**Table 2.** OBO Foundry principles and their automated checks

Principle	Summary	Automated check
Open (http://obofoundry.org/principles/fp-001-open.html)	The ontology MUST be openly available to be used by all without any constraint other than (a) its origin must be acknowledged and (b) it is not to be altered and subsequently redistributed in an altered form under the original name or with the same identifiers.	The registry data entry is validated with JSON Schema. The license schema ensures that a license entry is present and that the entry has a URL and label. The schema also checks that the license is one of the CC0 or CC-BY licenses. Then, annotations from the ontology are retrieved and the ‘dcterms:license’ annotation is retrieved (if exists). The script ensures that the correct ‘dcterms:license’ property is used and compares this license to the registry license to ensure that they are the same. Note that many ontologies currently fail this check due to discrepancies between the ontology file and the registry metadata, but we still require an ontology to conform to this principle in order to join the OBO Foundry.
Common Format (http://obofoundry.org/principles/fp-002-format.html)	The ontology is made available in a common formal language in an accepted concrete syntax.	The ontology is loaded using OWLAPI. If the ontology is successfully loaded, it is assumed that it is in a good format.
URI/Identifier Space (http://obofoundry.org/principles/fp-003-uris.html)	Each class and relation (property) in the ontology must have a unique URI identifier that follows the format: a base URI + a prefix that is unique within the Foundry + a local numeric identifier.	All entity IRIs are retrieved from the ontology, excluding annotation properties. Annotation properties may use hashtags and words due to legacy OBO conversions for subset properties. All other IRIs are checked if they are in the ontology’s namespace. If the IRI begins with the ontology namespace, the next character must be an underscore. The IRI is also compared to a regex pattern to check if the local ID after the underscore is numeric.
Versioning (http://obofoundry.org/principles/fp-004-versioning.html)	The ontology provider has documented procedures for versioning the ontology, and different versions of ontology are marked, stored and officially released.	The version IRI is retrieved from the ontology and, if found, this IRI is compared to a regex pattern to determine if it is in date format.
Scope (http://obofoundry.org/principles/fp-005-delineated-content.html)	The scope of an ontology is the extent of the domain or subject matter it intends to cover. The ontology must have a clearly specified scope and content that adheres to that scope.	First, the registry data is checked for a ‘domain’ tag. If it is present, the domain is compared to all other ontology domains. If the ontology shares a domain with one or more other ontologies, we return a list of those ontologies.
Textual Definitions (http://obofoundry.org/principles/fp-006-textual-definitions.html)	The ontology has textual definitions for the majority of its classes and for top-level terms in particular.	ROBOT ‘report’ is run over the ontology. A count of violations for each of the following checks is retrieved from the report object: duplicate definition, multiple definitions and missing definition. The ROBOT report will warn on any and all missing definitions, so in order to pass this check, all terms in an ontology must have distinct textual definitions.
Relations (http://obofoundry.org/principles/fp-007-relations.html)	Relations should be reused from the Relations Ontology (RO).	The object and data properties from the ontology are compared to existing RO properties.
Documentation (http://obofoundry.org/principles/fp-008-documented.html)	The owners of the ontology should strive to provide as much documentation as possible. The documentation should detail the different processes specific to an ontology life cycle and target various audiences (users or developers).	The registry data is checked for ‘homepage’ and ‘description’ entries. If the homepage is present, the URL is checked to see if it resolves (does not return an HTTP status of 400 or greater).
Documented Plurality of Users (http://obofoundry.org/principles/fp-009-users.html)	The ontology developers should document that the ontology is used by multiple independent people or organizations.	The registry data is checked for ‘usages’ entries.
Commitment to Collaboration (http://obofoundry.org/principles/fp-010-collaboration.html)	OBO Foundry ontology development, in common with many other standards-oriented scientific activities, should be carried out in a collaborative fashion.	N/A—this cannot be automated at this time. This principle does not appear in any dashboard result.
Locus of Authority (http://obofoundry.org/principles/fp-011-locus-of-authority.html)	There should be one person responsible for communications between the community and the ontology developers, for communicating with the Foundry on all Foundry-related matters, for mediating discussions involving maintenance in the light of scientific advance and for ensuring that all user feedback is addressed.	The registry data entry is validated with JSON Schema to ensure that a contact entry is present and that the entry has a name and email address.
Naming Conventions (http://obofoundry.org/principles/fp-012-naming-conventions.html)	Each entity within the ontology must have a unique label and must not have more than one label. All labels should be declared using the ‘rdfs:label’ property.	ROBOT ‘report’ is run over the ontology. A count of violations for each of the following checks is retrieved from the report: duplicate label, multiple labels and missing label.
Maintenance (http://obofoundry.org/principles/fp-016-maintenance.html)	The ontology needs to reflect changes in scientific consensus to remain accurate over time.	A version Internationalized Resource Identifier (IRI) is retrieved from the ontology and checked against a regex pattern to determine if it is in date format. If so, the date is retrieved to ensure that the ontology is updated in a timely manner. While regular releases are a good indicator of maintenance, we realize that this does not necessarily mean that the ontology is up to date with scientific consensus. At this time, we do not have the methods to fully validate this principle as it is written.
Responsiveness (http://obofoundry.org/principles/fp-020-responsiveness.html)	The ontology developers must offer a channel for community participation in the form of suggestions and requests.	The registry data is checked for a ‘tracker’ entry.

**Table 3.** OBO Foundry principles and their GitHub issues for discussion of automated validation

Principle	Automated validation GitHub issue
Open	https://github.com/OBOFoundry/OBOFoundry.github.io/issues/1019
Common Format	https://github.com/OBOFoundry/OBOFoundry.github.io/issues/1018
URI/Identifier Space	https://github.com/OBOFoundry/OBOFoundry.github.io/issues/1017
Versioning	https://github.com/OBOFoundry/OBOFoundry.github.io/issues/1016
Scope	https://github.com/OBOFoundry/OBOFoundry.github.io/issues/1015
Textual Definitions	https://github.com/OBOFoundry/OBOFoundry.github.io/issues/1010
Relations	https://github.com/OBOFoundry/OBOFoundry.github.io/issues/981
Documentation	https://github.com/OBOFoundry/OBOFoundry.github.io/issues/1009
Documented Plurality of Users	https://github.com/OBOFoundry/OBOFoundry.github.io/issues/1008
Commitment to Collaboration	N/A—this principle cannot be automatically validated at this time
Locus of Authority	https://github.com/OBOFoundry/OBOFoundry.github.io/issues/1007
Naming Conventions	https://github.com/OBOFoundry/OBOFoundry.github.io/issues/1006
Maintenance	https://github.com/OBOFoundry/OBOFoundry.github.io/issues/1020
Responsiveness	https://github.com/OBOFoundry/OBOFoundry.github.io/issues/959

### Establishing a framework for automatic evaluation of ontology metadata

In order to semi-automate the process of determining ontology conformance, we implemented a validation suite that displays its results through the OBO dashboard (http://dashboard.obofoundry.org/dashboard/index.html). The dashboard implements an executable programmatic expression of each principle and a framework for running these checks and for delivering a web-based report. The dashboard is implemented on top of the ROBOT software suite (12) and, in particular, uses the ability of ROBOT to reason over ontologies and to generate detailed reports. Additionally, the validation suite checks the metadata for each ontology in the OBO registry. For example, the curated ‘usages’ tag is used to determine if the ontology fulfills the criterion for having a plurality of independent users.

**Figure 2.**
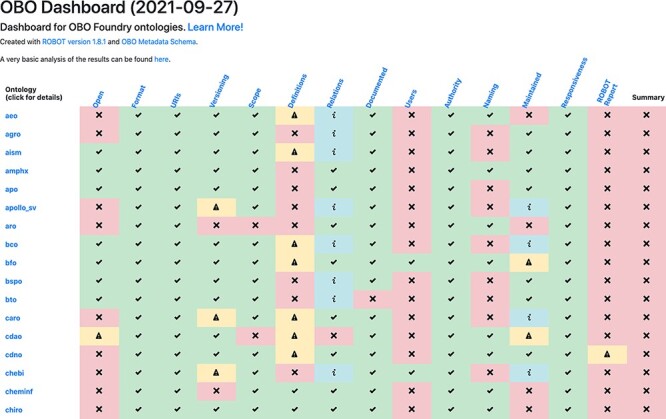
The OBO dashboard (truncated). The rows represent OBO ontologies (of which the first 15 in alphabetical order are shown here) and the columns are the OBO principles. The final column, ‘Summary’, shows whether the ontology passed all of the tests. Clicking on the ontology ID in the far left column directs to a detailed report page.

The dashboard results are shown as a grid where each ontology is a row and each OBO principle a column, with each cell indicating results of the check for this combination (Figure 2). For each OBO principle, the dashboard links to (i) the web page for that principle, which links to (ii) a web page describing the automated test, which links to (iii) a tracker issue for the automated test. Each ontology has a detailed report page accessible from the main dashboard by clicking on the ontology ID. This provides a breakdown of the problems encountered and suggestions on how to fix them.

**Figure 3.**
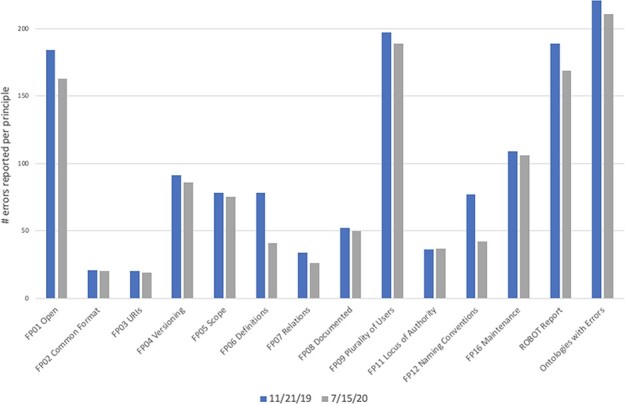
Number of errors reported by dashboard on 11 November 2019 (blue bars) and 15 July 2020 (gray bars). The final column, ‘Ontologies with Errors’, is the total number of ontologies that had one or more errors, not a count of all errors. While more ontologies joined the OBO Foundry between these two dates, we only included statistics for the 223 ontologies that were present and active in both the first run and the second run. The automated checks remained the same during this time period.

When a preliminary version of the dashboard was first announced to the OBO ontology maintainers in early 2020, several ontology maintainers started fixing the problems identified in the dashboard scripts. Specifically, comparing the experimental dashboard runs in 11/2019 (prior to the announcement of the OBO dashboard work) vs. 07/2020, we found a significant reduction in reported errors when doing a pairwise comparison for each error type identified by the dashboard code before and after the introduction of the dashboard (*P* = 0.0005, Wilcoxon test (13), Figure 3). This pairwise comparison was limited to the set of unlinked data, which does not include numbers from ‘ROBOT Report’ or ‘Ontologies with Errors’ from Figure 3.

At the same time, users reported issues with the automated validation code leading to false-positive and false-negative results, which were subsequently fixed and have led to the more robust version of the code implemented in the current version of the dashboard. While the iterative updates to the code mean that current numbers of validation issues cannot be compared to those at the start of the project, the community engagement and the noticeable drop in issues between versions that could be compared demonstrate that the OBO ontology developer community is responsive to the issues identified by the dashboard and that highlighting problems in a transparent manner can be a productive first step toward resolving them.

**Figure 4.**
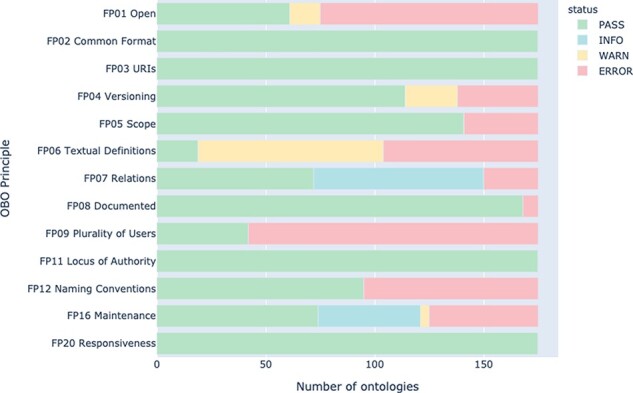
Summary of principle conformance across all active OBO Foundry ontologies in May 2021.

As can be seen in Figure 4, as of May 2021, four principles were fully conformed to by all 175 active OBO Foundry ontologies: ‘FP02 Common Format’, ‘FP03 URIs’, ‘FP11 Locus of Authority’ and ‘FP20 Responsiveness’. The principle that was least conformed to was ‘FP06 Textual Definitions’, with only 19 ontologies (about 11%) fully passing this check. Note that ‘FP20 Responsiveness’ was added to the OBO Foundry in March 2021, so the numbers in Figure 3 do not include this check. Additionally, Figure 3 includes results for the 223 OBO Foundry ontologies that were active during the Nov 2019 and July 2020 runs, whereas Figure 4 only includes results for ontologies that were active during the May 2021 run.

## Discussion

The scientific community has always relied on sharing data through publications or personal communications. The recently developed FAIR principles (14) spell out what it takes for shared data to be findable, accessible, interoperable and reproducible. A key requirement of FAIR is to use vocabularies that are reusable across projects, which aligns with the original goals of the OBO project, which precedes the formulation of the FAIR principles by more than a decade. Thus, the goals of OBO and FAIR are highly compatible, and there is no conflict between these principles. The lessons learned from our work on OBO should be taken into consideration when evaluating FAIR principles.

Like FAIR, the original OBO principles served as a rallying cry, galvanizing a community to work toward a broadly articulated vision. After two decades of work on OBO, we found that relying on human review of such principles is difficult to standardize and does not scale. Instead, we decided to turn each principle into operational tests for conformance. We found that this process was beneficial to communicating clearly what each principle was meant to accomplish and to provide clear guidance for ontology developers on what they needed to do to achieve compliance with the principle.

Going forward, we plan to run the OBO dashboard on all new ontologies requesting OBO membership and on each new release of every OBO member project. Given the free availability of the code, it can be run (and in some cases already is running) as part of internal ontology development pipelines to test internal release candidates. We expect that this process will identify weaknesses in the current pipeline and result in continuous improvements of the tests themselves and of the shared understanding of what the tests (and the principles) are meant to achieve across the OBO community.

There are several limitations to our approach that suggest paths for future work. First, the current framework examines a single ontology at a time. We are planning to extend the checks to run across sets of ontologies to provide insights on inter-ontology consistency. Second, not all principles formulated for the OBO Foundry can be checked reliably in an automated fashion. Specifically, human review is needed to check for scope, a plurality of users and cooperation with existing ontologies. While these limitations have to be kept in mind, it is important to realize how much more consistent and up to date the current automated system is compared to the previous practice of relying on manual human volunteer reviewers. Furthermore, we want to better align with the existing World Wide Web Consortium (W3C) and other standards where appropriate. One area we plan to improve is handling versioning in the OBO ontologies, for which many standards already exist.

In conclusion, this manuscript highlights the OBO dashboard and associated automated test as the main advancement of the OBO Foundry in 2021. As this is the first official publication of the OBO dashboard, we expect that there will be community feedback and criticism on the specific implementation of the checks implemented, and we very much welcome that. We hope that the quantitative nature of the dashboard and its underlying automated rules will make these discussions constructive. Furthermore, we hope that other standardization-focused projects will take inspiration from the OBO Foundry’s successful effort to assess and quantify our evaluation principles and will adopt similar standards and methods for reviewing, as has already happened with the AgroPortal (7) and semantic web for Earth and environmental terminology (15) communities.
